# The Relations Between Predatory Fungus and Its Rotifer Preys as a Noteworthy Example of Intraguild Predation (IGP)

**DOI:** 10.1007/s00248-019-01398-4

**Published:** 2019-06-24

**Authors:** Edyta Fiałkowska, Wojciech Fiałkowski, Agnieszka Pajdak-Stós

**Affiliations:** Faculty of Biology, Institute of Environmental Sciences, Aquatic Ecosystems Group, Jagiellonian University, ul. Gronostajowa 7, 30-387 Kraków, Poland

**Keywords:** *Cephalodella gibba*, Competition, *Lecane inermis*, Predation, *Zoophagus* sp., Activated sludge

## Abstract

Intraguild predation (IGP) is a widespread interaction combining predation and competition. We investigated a unique IGP example among predacious fungus *Zoophagus* sp. and two rotifers, the predacious *Cephalodella gibba* and the common prey *Lecane inermis*. We checked the influence of the fungus on its competitor *C*. *gibba* and their joint influence on shared prey *L*. *inermis*, and the impact of the competitive predator on the growth of predacious fungus. The experiment on grown mycelium showed that *Zoophagus* strongly, negatively influences the growth of *C*. *gibba* (intermediate consumer) whose number did not increase throughout the experiment. The intermediate consumer was also trapped by *Zoophagus* and become extinct when it was its only prey, whereas in the absence of the fungus and with unlimited access to prey, its number grew quickly. As only few *C*. *gibba* were trapped by fungi when common preys were present, competition for food seems to have stronger effect on intermediate consumer population than predation. The experiment with conidia of the fungus showed that intermediate consumer significantly limits the growth of *Zoophagus* by reducing the number of available prey. It was observed that although the fungus can trap *C*. *gibba*, the latter does not support its growth. Trapping the intermediate consumer might serve to eliminate a competitor rather than to find a source of food. The chances of survival for *L*. *inermis* under the pressure of the two competing predators are scarce. It is the first example of IGP involving representatives of two kingdoms: Fungi and Animalia.

**Electronic supplementary material:**

The online version of this article (10.1007/s00248-019-01398-4) contains supplementary material, which is available to authorized users.

## Introduction

The feeding interdependence has long been recognized as crucial for forming the structure of ecological communities. The history of research concerning relationships such as interspecific competition goes back to the first half of the twenty-first century [1] and literature therein]. Also, the subject of predation and its impact on competition has been intensely studied both theoretically and practically in various habitats [2–5]. Even though predation and competition occur simultaneously in ecological systems, they were often regarded separately [6]. Another type of interaction combining competition and predation is intraguild predation (IGP) defined as “the killing and eating of species that use similar, often limiting resources, and are thus potential competitors” [7]. This kind of interaction occurs among the members of the same “guild” which according to the definition given by Root [8] is a group of species that exploit the same class of environmental resources in a similar way.

**Fig. 1:**
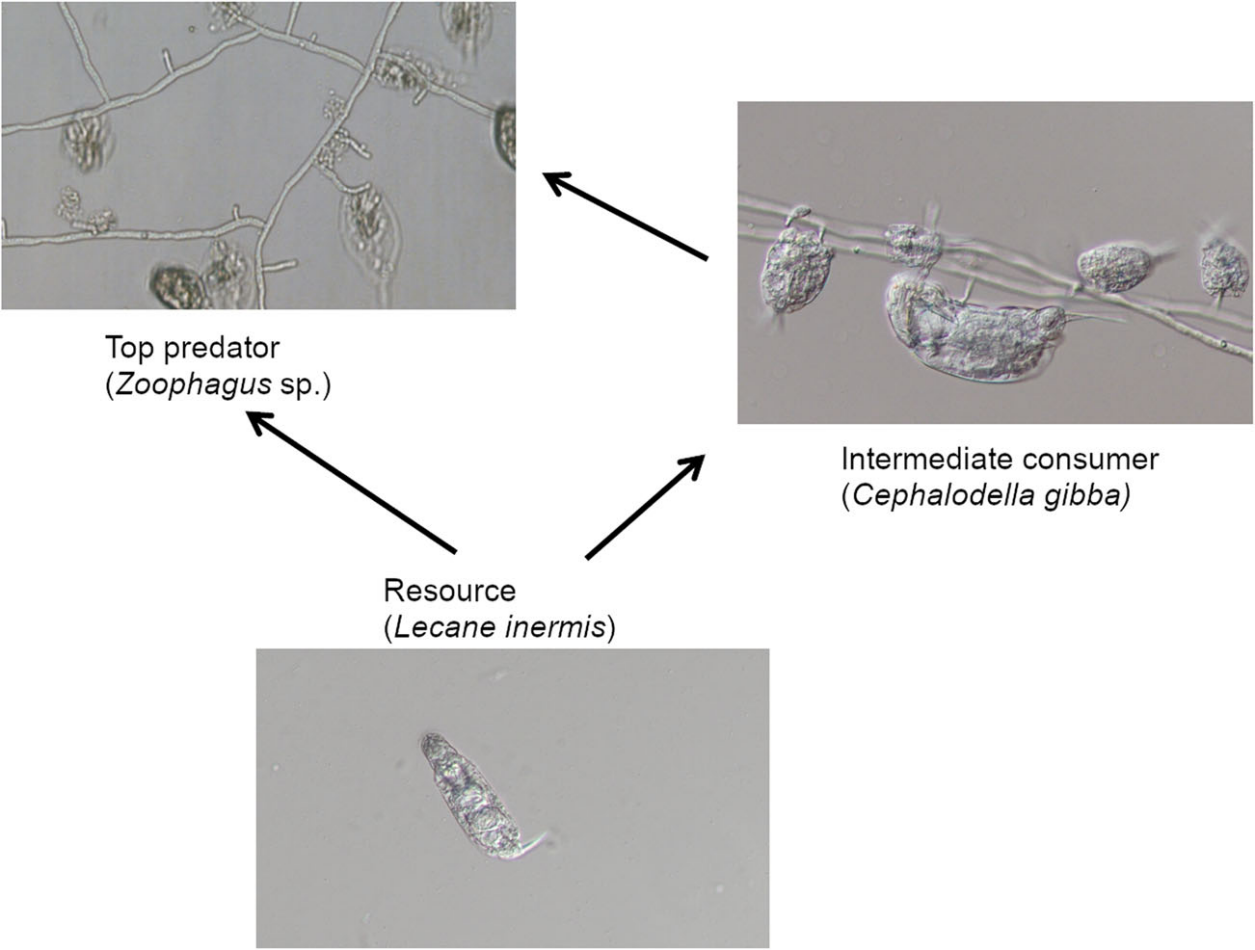
The scheme of a three-species food web with intraguild predation (IGP) where top predator (left) feeds on resources (bottom) as well as on an intermediate consumer (right – the biggest individual). The picture on the right depicts both *C*. *gibba* and *L*. *inermis* caught by the fungus

As the factor significantly influencing occurrence, abundance, distribution, and evolution of many species, the IGP was thoroughly investigated in many different ecosystems [7, 9, 10]. The research of IGP among freshwater organisms was conducted, among others, on dragonfly larvae [11], protists [12, 13], cyanobacteria and chrysophytes [14], and also theoretically on bacteria and viruses [15]. In the course of our research on the functioning of activated sludge community, we found an interesting example of IGP interaction among two species of rotifers (*Cephalodella gibba* and *Lecane inermis*) and a predatory fungus (*Zoophagus* sp.). Both species of rotifers were reported to occur in activated sludge [16–19]. *L*. *inermis* is well documented to feed on biofilm, unicellular, and filamentous bacteria [20–23], whereas the data concerning *C*. *gibba* are limited. It was reported that a species of *Cephalodella* feed on mixotrophic flagellates [24] and *C*. *gibba* occurring in freshwater streams feeds on biofilm, but it can also prey on other rotifers [25]. Our observations revealed that *C*. *gibba* is voracious and feeds readily on *L*. *inermis*, which is also a prey of *Zoophagus* sp. Some strains of *Zoophagus* were reported to trap loricated rotifers, among them *L*. *inermis* [26–28]. As the fungus and *C*. *gibba* exploit the same resource, they can be treated as a “guild” and *L*. *inermis* can be treated as shared resource for both predatory organisms. Moreover, we observed that the fungus also traps *Cephalodella* rotifers. Thus, the relationship seems to follow the pattern of a basic three-species IGP where top predator (the fungus) feeds on resource (*L*. *inermis*) and on the intermediate consumer (*C*. *gibba*) (Fig. 1). Even though many examples of IGP exist and were investigated, to our knowledge, there are no researches on IGP systems in which a predatory fungus is the top predator.

To investigate the reciprocal relationship between the three species, we conducted two sets of experiments. The first experiment tested the impact of the predatory fungus on *C*. *gibba* growth rate on the one hand, and the joint impact of both predatory organisms on their shared prey, on the other. The second experiment tested the influence of competitive predator’s presence on the growth of *Zoophagus* sp.

## Material and Methods

### Cultures

All the organisms used in the experiments were isolated from activated sludge. *L*. *inermis* (clone 1.A2.15), used as prey organism, were isolated from a municipal wastewater treatment plant (WWTP) in the Silesia region in Poland in 2015.The clone was obtained from a single individual transferred with a micropipette from a sludge sample to a separate vessel filled with Żywiec brand spring water and fed with NOVO (nutrition powder used for rotifer mass culture, patent EP2993978(A1)) [29]. The cultures are maintained in darkness at 20 °C.

The clone of *Cephalodella* sp. and the strain of *Zoophagus* sp. were isolated in 2018 from a pharmaceutical WWTP situated in the north-west of Poland. The clone of *Cephalodella* sp., coded CK1, was obtained from a single individual transferred to a separate well filled with Żywiec brand spring water and fed with *L*. *inermis*. Even though *Cephalodella* feeds readily on *L*. *inermis*, the species is omnivorous. Apart from alive *L*. *inermis*, it also ingests dead individuals and unicellular green algae. The specimens are 227–237-μm long (with toes); the toes are 50–73-μm long. The species of rotifers were identified on the basis of morphological features [30, 31].

Pieces of fungal mycelium were transferred from a sludge sample to Petri dishes filled with Żywiec spring water. *Lecane* rotifers were added as a food source. When the fungus produced conidia, they were transferred individually to separate wells in culture test plates and maintained in darkness at 20 °C. One of the strains, coded as POL1, was used for the experiments. We classified this fungus as *Zoophagus* according to a key by Dick [32], in which the main criterion distinguishing *Zoophagus* from *Lecophagus* is the septation of the mycelium. The mycelium of the fungus is non-septate and branched with lateral branches growing more or less perpendicularly to the main mycelium (Fig. 1). The width of the mycelium varies between 3.6 and 10.4 μm. Adhesive pegs are perpendicular to the mycelium, spaced irregularly at intervals from 6 to 100 μm. There are also very long fragments, especially of newly grown mycelium, devoid of any trapping pegs. The length of the adhesive pegs highly varied (16–75 μm). In some cases, the trapping devices are branched; in other, a prey seems to be caught at the tip of a growing mycelium. Conidia are solitary, non-septate, and oblong, each separated from the mycelium by attenuated isthmus. They rarely get fully separated from the mycelium and start to germinate when still connected to it. It is often observed that several conidia grow linearly one after another, separated by contractions between them. However, the conidia become easily separated when some movement of the medium occurs, e.g., by swirling of the Petri dish. The length of a solitary conidium varies from 200 to 310 μm, and the width in the thickest part between 7.5 and 8.9 μm.

**Fig. 2:**
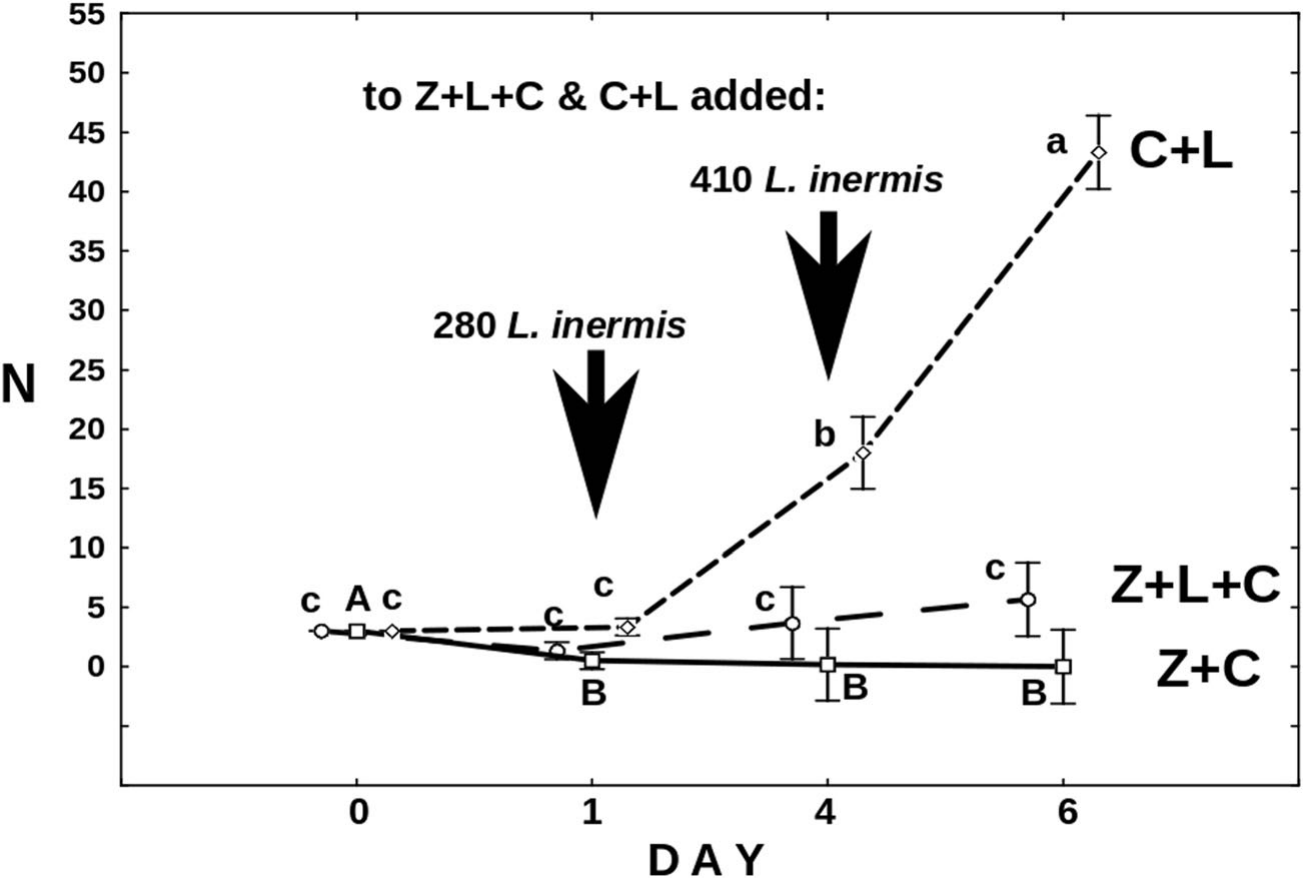
The mean number of active *Cephalodella gibba* on consecutive days of the experiment in the following treatments: Z+L+C (*Zoophagus* sp., *L*. *inermis*, and *C*. *gibba*), Z+C (*Zoophagus* sp. and *C*. *gibba*), and C+L (*C*. *gibba* and *L*. *inermis*). Black arrows indicate the dates when prey rotifers were introduced. Points sharing any common letter do not differ significantly (*p* > 0.05). The whiskers indicate confidence intervals

### Experiment I

The experiment was conducted in 24-well tissue plates. Into each of 18 wells, three conidia of the fungus were transferred with a micropipette. One milliliter of Żywiec water was added into each well and then 25 μL of dense *L*. *inermis* culture (containing approximately 300 individuals) was inoculated, so that the fungus would start growing. Every second day, we checked each well if there still were alive rotifers, available for the fungus. On the seventh day, when there were only single alive *Lecane* in the wells, with the help of micropipette, we exchanged the medium in the wells so that all the *Lecane* were removed. One milliliter of fresh Żywiec water was added to each well. Then, 30 wells were divided into 5 experimental groups:

$$ \mathrm{Z}+\mathrm{L}+\mathrm{C};\mathrm{Z}+\mathrm{C};\mathrm{C}+\mathrm{L};\mathrm{Z}+\mathrm{L};\mathrm{L},\mathrm{each}\ \mathrm{with}\ 6\ \mathrm{replicates}, $$

where:

Z: mycelium of *Zoophagus*,
C: three individuals of *Cephalodella*,
L: approximately 240 individuals of *Lecane* delivered in 25 μL of dense culture.

The wells were incubated in darkness at 20 °C. Twenty four hours after the start of the experiment, we counted the number of free-swimming *Cephalodella* and of those trapped by the fungus. We also counted the number of laid eggs. As in the wells with *Zoophagus*, most of the rotifers were already trapped by the fungus; we added 25 μL of dense culture of *Lecane* containing approximately 280 individuals to each well with *Lecane* rotifers. The counting was repeated 3 days (72 h) later. Again, 25 μL of dense *Lecane* culture containing approximately 410 individuals was added to each well. This time, the number of *Lecane* was higher to meet the requirements of growing *Zoophagus* mycelium as well as the increasing number of *Cephalodella*. The counting was repeated again after the following 48 h. This time, active *Lecane* rotifers were counted in Z+L, C+L trials, and in control (L).

### Experiment II

The experiment was conducted in 30 wells of 24-well tissue plates. Each well was filled with 1 mL of Żywiec water. Into each of 18 wells, three conidia of the fungus were transferred with a micropipette. Comparatively low number of conidia made it possible to evaluate the growth of the fungus. The conidia were measured and the total length of all the three conidia in each well was calculated. Then, the wells were divided into 5 experimental groups:

$$ \mathrm{Z}+\mathrm{L}+\mathrm{C};\mathrm{Z}+\mathrm{C};\mathrm{Z}+\mathrm{L};\mathrm{L};\mathrm{C},\mathrm{each}\ \mathrm{with}\ 6\ \mathrm{replicates}, $$

where:

Z: three conidia of *Zoophagus*,
C: three individuals of *Cephalodella* in the Z+L+C treatment or ten *Cephalodella* in the Z+C and C treatments,
L: approximately 210 individuals of *Lecane* delivered in 25 μL of dense culture.

Approximately 210 *Lecane* individuals were inoculated into 12 wells with the fungus (Z+L+C, Z+L) as well as into six wells serving as control (L). To the wells with the fungus and *Lecane*, we added three *Cephalodella* rotifers (Z+L+C). Into each of the remaining six wells with fungus conidia, we inoculated 10 *Cephalodella* rotifers (Z+C). To the last six wells serving as *Cephalodella* control, we added 10 rotifer individuals (C). The wells were incubated in darkness at 20 °C.

After 24 and 48 h, we measured the length of each conidium together with growing mycelium, and the total length for each well was calculated. We also counted the number of trapped and active *Cephalodella* and the number of caught *Lecane* individuals. The measurements were repeated after the following 24 h, but this time, the length of the fungus was measured only in the Z+C trial, as the mycelium in the trials with *Lecane* was already too long to be measured precisely. As the number of *Cephalodella* in Z+C trial was very low, 10 additional individuals were inoculated. In all trials with *Lecane*, 25 μL of dense culture containing approximately 320 individuals was added into each well to make sure that there was a surplus of the prey.

After the following 3 days, the numbers of active and trapped *Cephalodella* in Z+L+C and Z+C and active *Cephalodella* in C trials were counted. At that time, also the number of active *Lecane* was counted in Z+L+C and Z+L trials. To check if there were any differences in the growth of fungus in Z+L+C and Z+L trials, we took three pictures from each well so that the “maternal” conidium was in the center of the field of view. Then, we measured the length of the fungus in each picture and calculated total length of mycelium for each well. The measurements were done with the help of an inverted microscope Olympus IX 71 with NIS image analysis system.

### Data analysis

Mean growth rate of *Cephalodella* was calculated according to the following formula:

$$ r=1/t\left(\ln \left({N}_t\right)\hbox{--} \ln \left({N}_0\right)\right) $$

where *t* is the day of the experiment and *N* is the number of rotifers.

In the first experiment, the significance of differences in the numbers of *Cephalodella* and/or its eggs between days in each treatment separately was tested by means of repeated measures ANOVA [33] followed by the Tukey post hoc analysis. In the second experiment, differences in the growth of *Zoophagus* mycelium during the first 3 days were analyzed in the same way. In the case of the trials where the abundances of rotifers were differently manipulated (see above), the significance of changes in the number of *Cephalodella* or *Lecane* was analyzed separately for each treatment by a pairwise *t* test for dependent samples. Pairwise *t* test for independent samples was employed in analyzing the differences in mycelium length or rotifer numbers at the end of respective experiment. Computations were performed, and graphs were prepared using STATISTICA 12.5 package [34].

## Results

The results of the first experiment showed that both predators, the fungus *Zoophagus* sp. and the rotifer *C*. *gibba*, are extremely voracious and they compete for their common prey. Figure 2 shows the changes in the number of active *Cephalodella* depending on the presence of the competing predator. Since the fourth day of the experiment, the mean numbers of *C*. *gibba* in the treatments with *Lecane* differed significantly (*F*_6,45_ = 155.50, *p* = 0.0). Without the presence of the fungus (C+L), *Cephalodella* began to rise sharply after the first day of the experiment, reaching the mean number of 43 and mean growth rate *r* = 0.45/day. In the treatment where all three organisms co-occurred, the fungus did not killed all the *Cephalodella* but its growth was strongly limited. On the last day of the experiment, average number of active individuals reached five, which means mean growth rate at the level of 0.11. Thus, the number of *Cephalodella* competing with predatory fungus at the end of the experiment was slightly higher in comparison with the start, but the difference was not statistically significant. As Fig. 2 shows, no *Cephalodella* individual survived to the end of the experiment in the treatment where no prey organisms were provided.

**Fig. 3:**
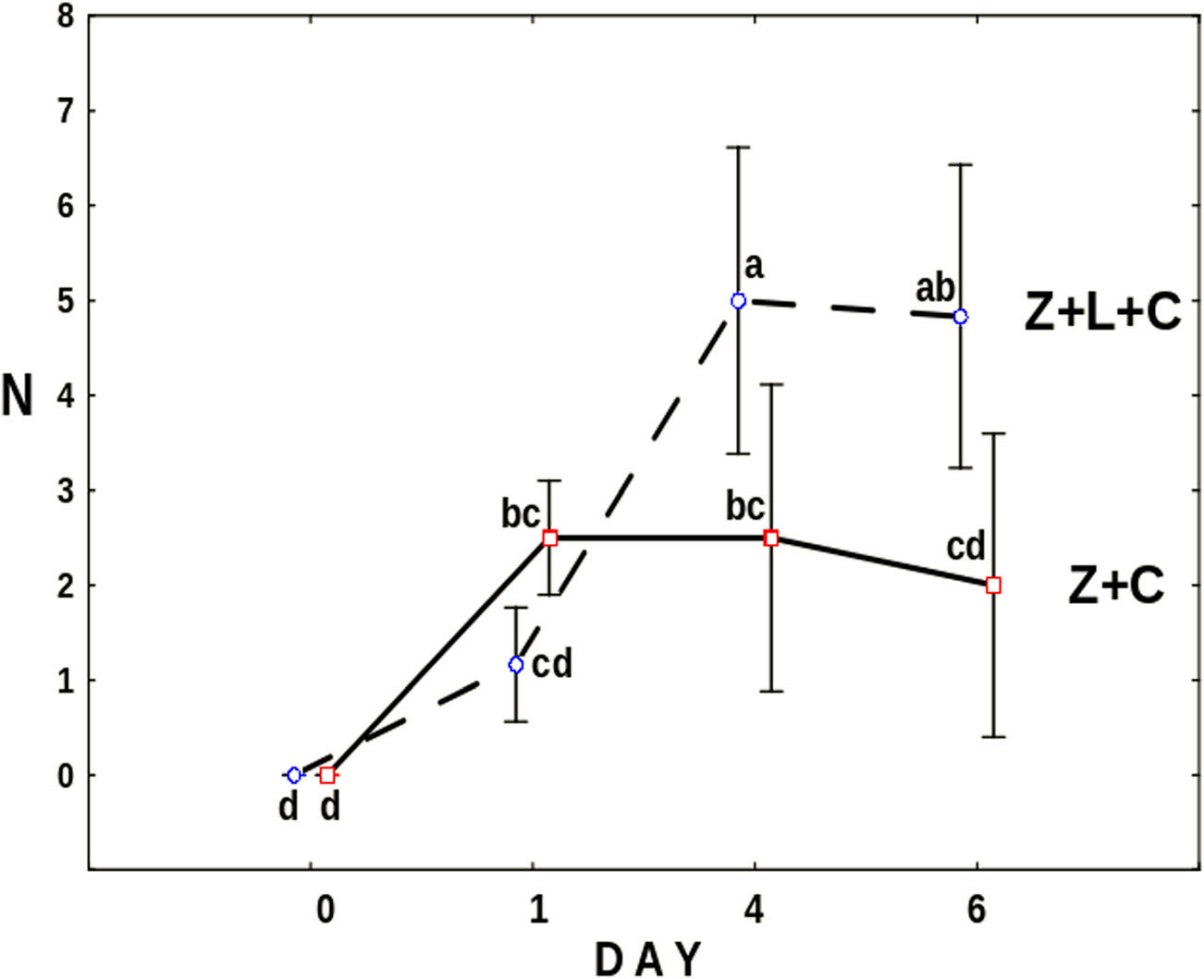
The mean number of *C*. *gibba* rotifers trapped by the fungus. Points sharing any common letter do not differ significantly (*p* > 0.05)

Figure 3 shows the number of *Cephalodella* individuals trapped by the fungus. On average, already within the first day after release, two out of three *C*. *gibba* were trapped by the fungus. This number was clearly, albeit not significantly, lower in the presence of *Lecane*. The effect of the latter became more conspicuous and significant (*F*_3,30_ = 9.48, *p* = 0.001) since the fourth day of the experiment when the mean number of trapped *Cephalodella* reached five, whereas in the Z+C treatment, it was about two.

**Fig. 4:**
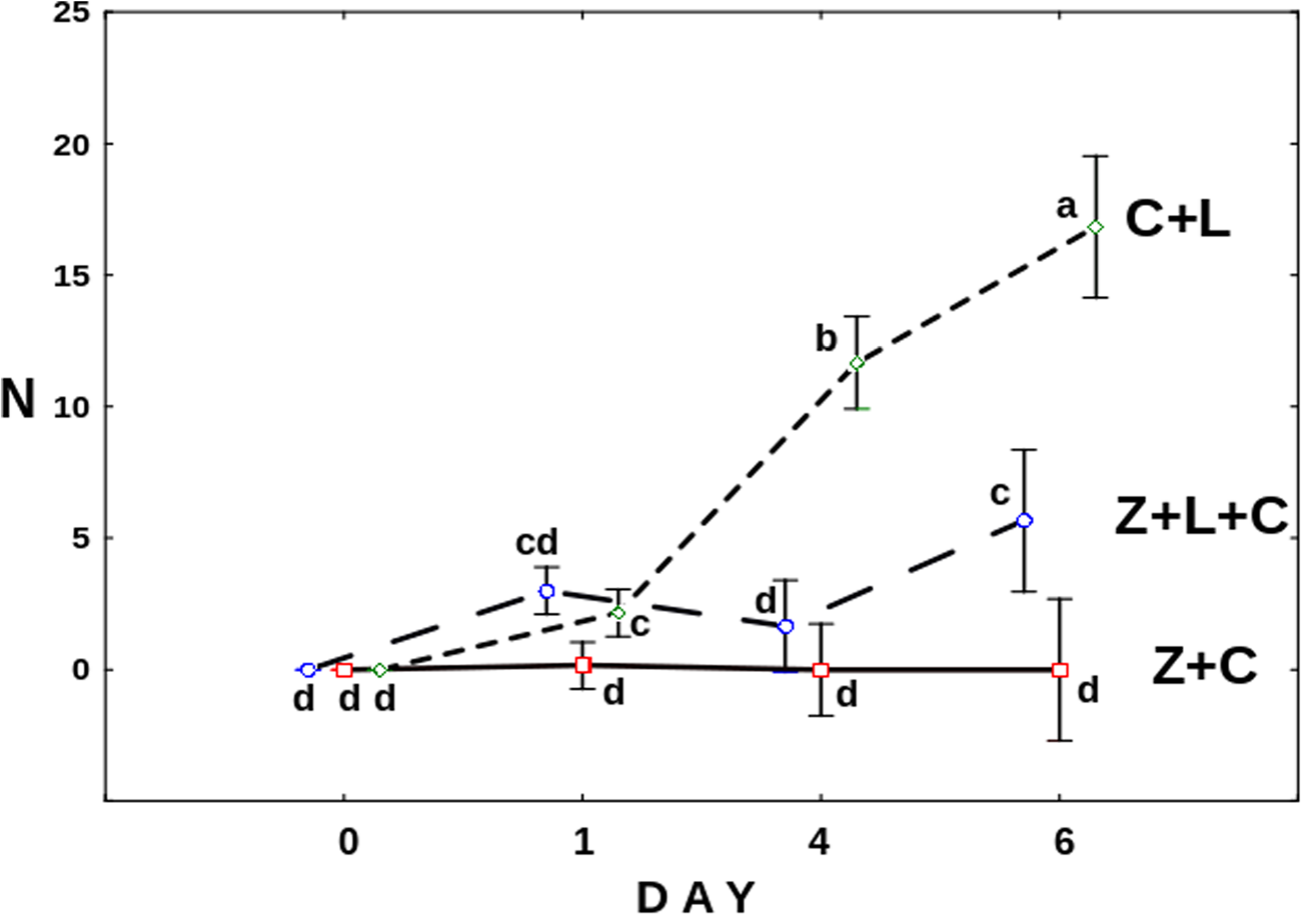
The mean number of *C*. *gibba* eggs on consecutive days in the following treatments: Z+L+C (*Zoophagus* sp., *L*. *inermis*, and *C*. *gibba*), Z+C (*Zoophagus* sp. and *C*. *gibba*), and C+L (*C*. *gibba* and *L*. *inermis*). Points sharing any common letter do not differ significantly (*p* > 0.05). The whiskers indicate confidence intervals

**Fig. 5:**
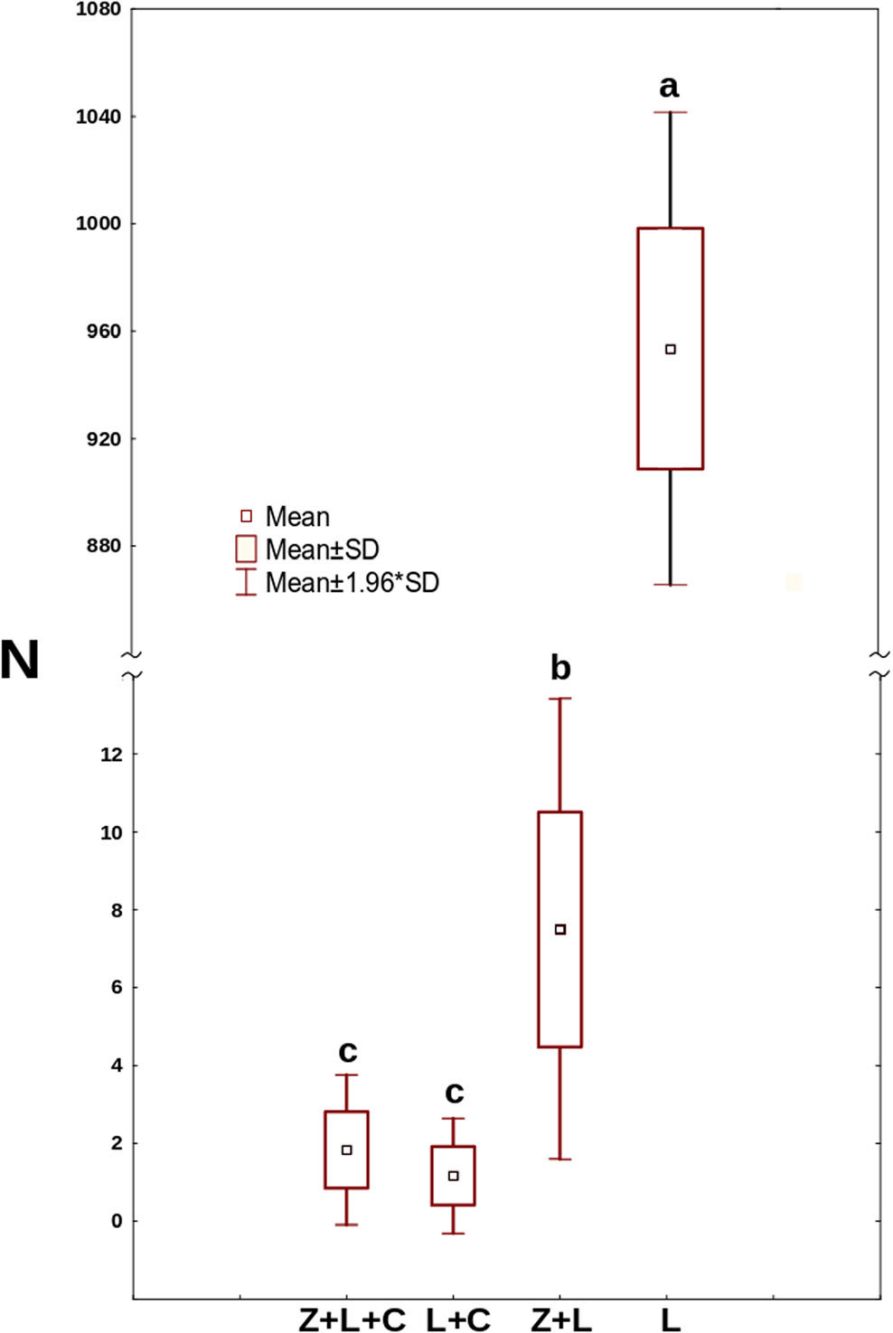
The mean number of active *L. inermis* rotifers at the end of the experiment. Points sharing any common letter do not differ significantly (*p* > 0.05)

The mean numbers of *C*. *gibba* eggs differed significantly between treatments (*F*_6,45_ = 39.405, *p* = 0.0). Apparently, in the presence of only *Lecane*, egg production in *C*. *gibba* was the highest (Fig. 4). From the second day onwards, the number of eggs on each day has been significantly higher than that on the preceding day. Addition of the fungus to the above setting clearly hampered egg production, which was especially pronounced since the fifth day of the experiment. Still, on the last day of the experiment, the number of eggs in Z+L+C was significantly higher than that in the Z+C treatment, in which *Cephalodella* ceased the production of eggs. The number of *L*. *inermis* at the end of the experiment was extremely low (Fig. 5). *Lecane* went almost extinct in the treatment with *Cephalodella* only and in the treatment with both predators. Although under the pressure of only the fungus (Z+L) the mean number of active *Lecane* was approximately four times higher in comparison with the C+L and Z+L+C treatments (Fig. 5), still the mean number of active rotifers was only eight. As approximate mean total number of *L*. *inermis* released into each well was about 930, the number of active rotifers at the end of the experiment was extremely low. To check if the mortality rate was not caused by starvation, on the last day of the experiment, we counted the *Lecane* rotifers in the L treatment, and mean number of alive rotifers reached 950 individuals.

**Fig. 6:**
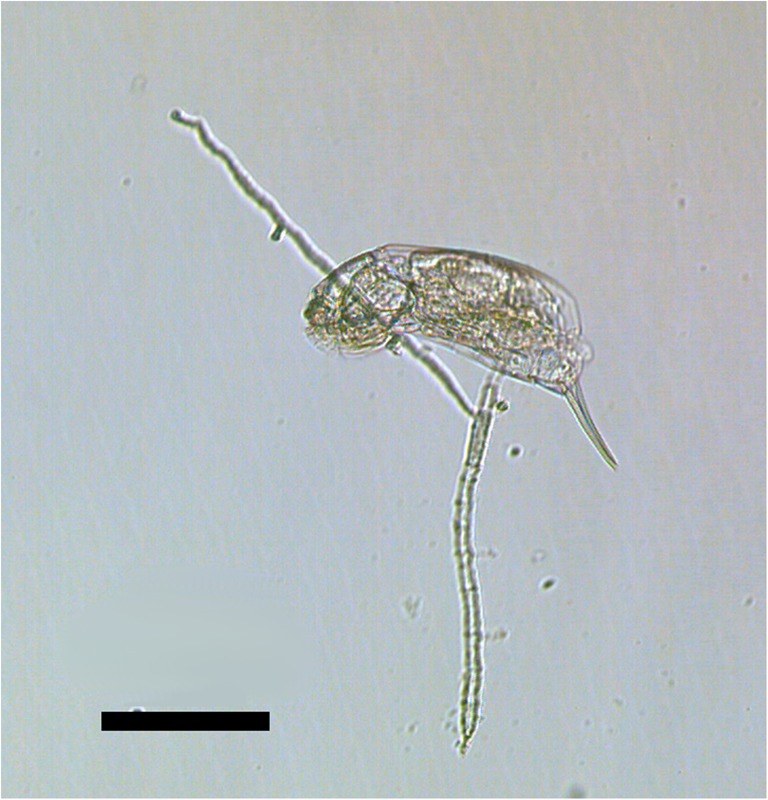
The conidium with freshly trapped *C*. *gibba*. The mycelium probably started to grow by withdrawing the cytoplasm from conidium. Scale bar indicates 100 μm

The second experiment in which predatory fungus was introduced in the form of conidia (three per well) showed that in the absence of *Lecane* (Z+C), conidia practically did not grow, even though single rotifer was trapped by them. Only one conidium out of 18 used in this treatment started to grow; however, it seems that the mycelium was supported by cytoplasm withdrawn from the conidium (Fig. 6) and not fed by the trapped *Cephalodella*. Later on the piece of mycelium degenerated. The growth of the fungus was limited in the Z+L+C treatment even though some of *Cephalodella* were eliminated by the fungus.

**Fig. 7:**
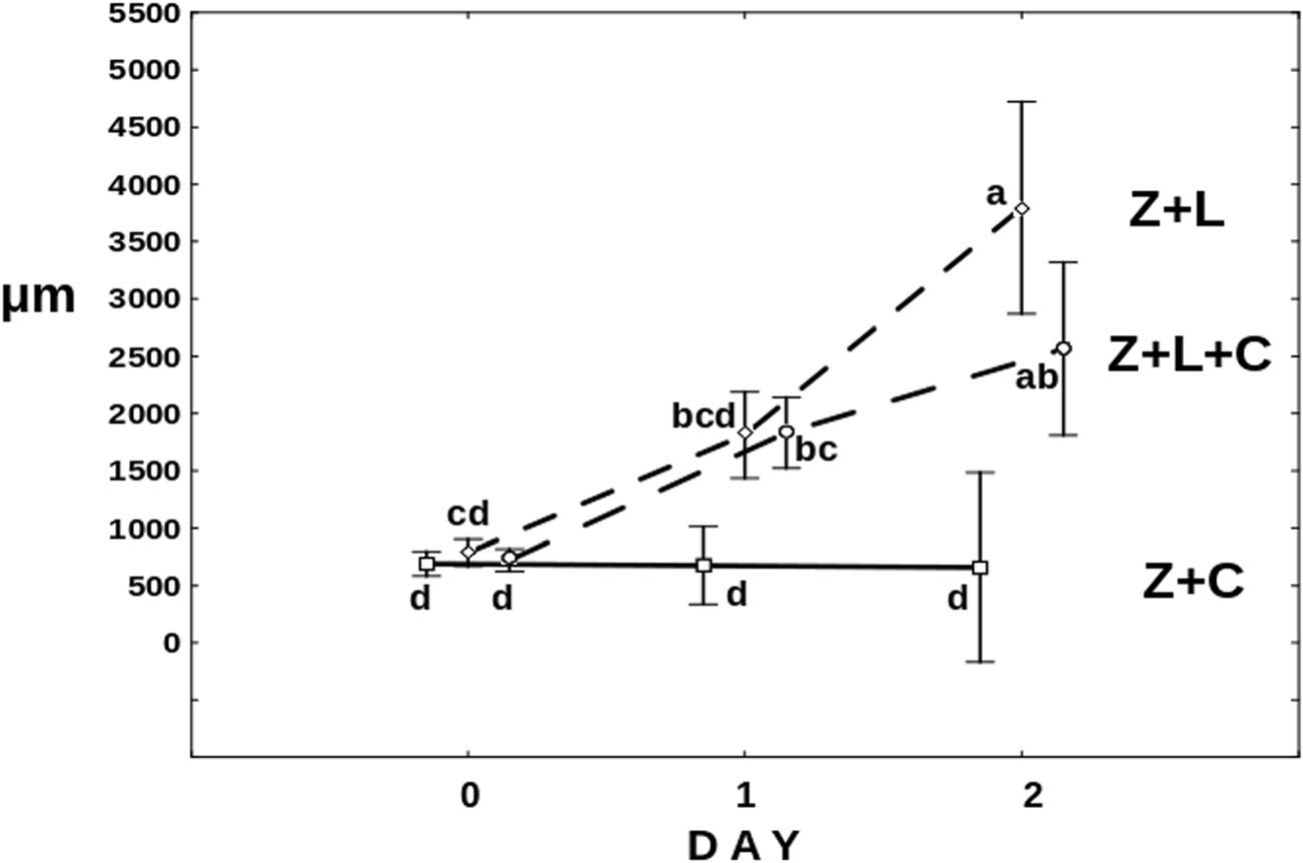
The mean total length of *Zoophagus* sp. mycelium in the Z+L, Z+L+C and Z+C treatments. Points sharing any common letter do not differ significantly (*p* > 0.05). Whiskers indicate confidence intervals

**Fig. 8:**
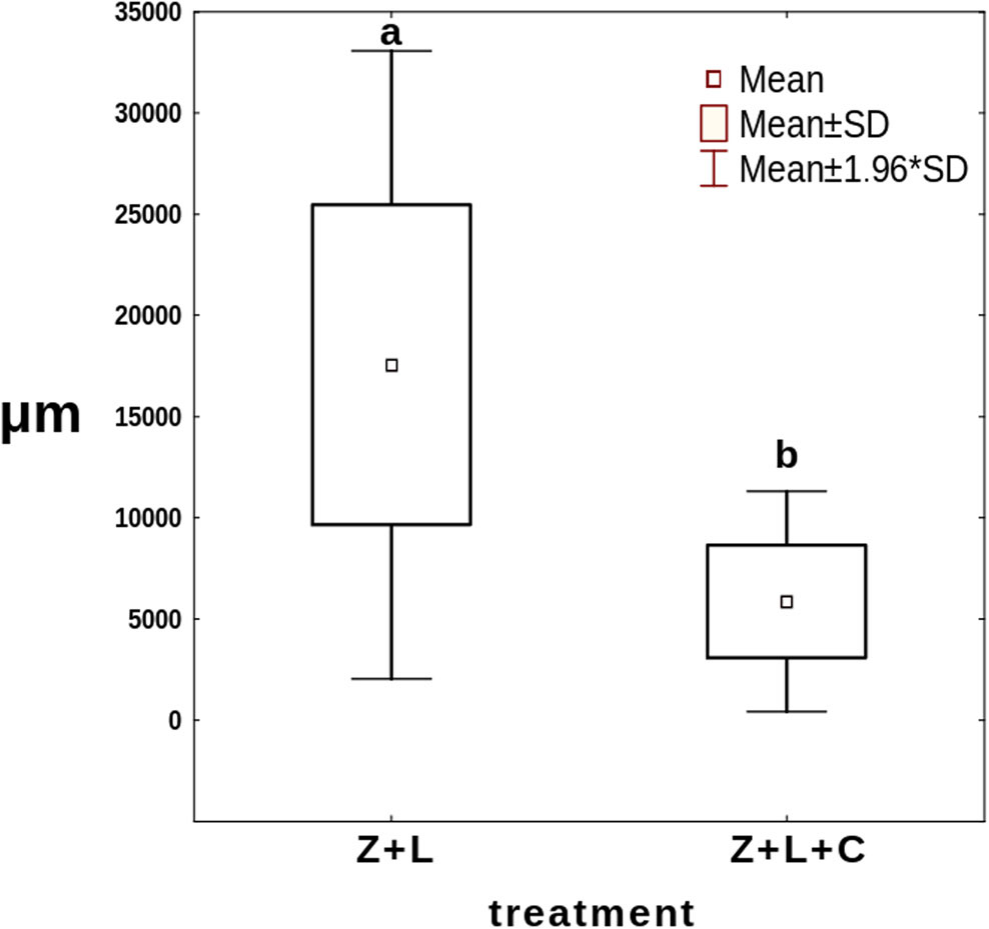
The mean length of *Zoophagus* sp. mycelium at the end of the experiment in the Z+L+C and Z+L treatments. Points sharing any common letter do not differ significantly (*p* > 0.05)

In the treatments with *Lecane* as a common prey (Z+L and Z+L+C), conidia started to grow already during the first 24 h. After the following 24 h, the growth of the fungus in the treatment without *Cephalodella* was apparently, albeit not significantly, quicker than that in the treatment where it was present (Fig. 7). At the end of the experiment, after a week, mycelium in the treatment without competitor was approximately three times longer than that in the treatment where it was present (Fig. 8).

**Fig. 9:**
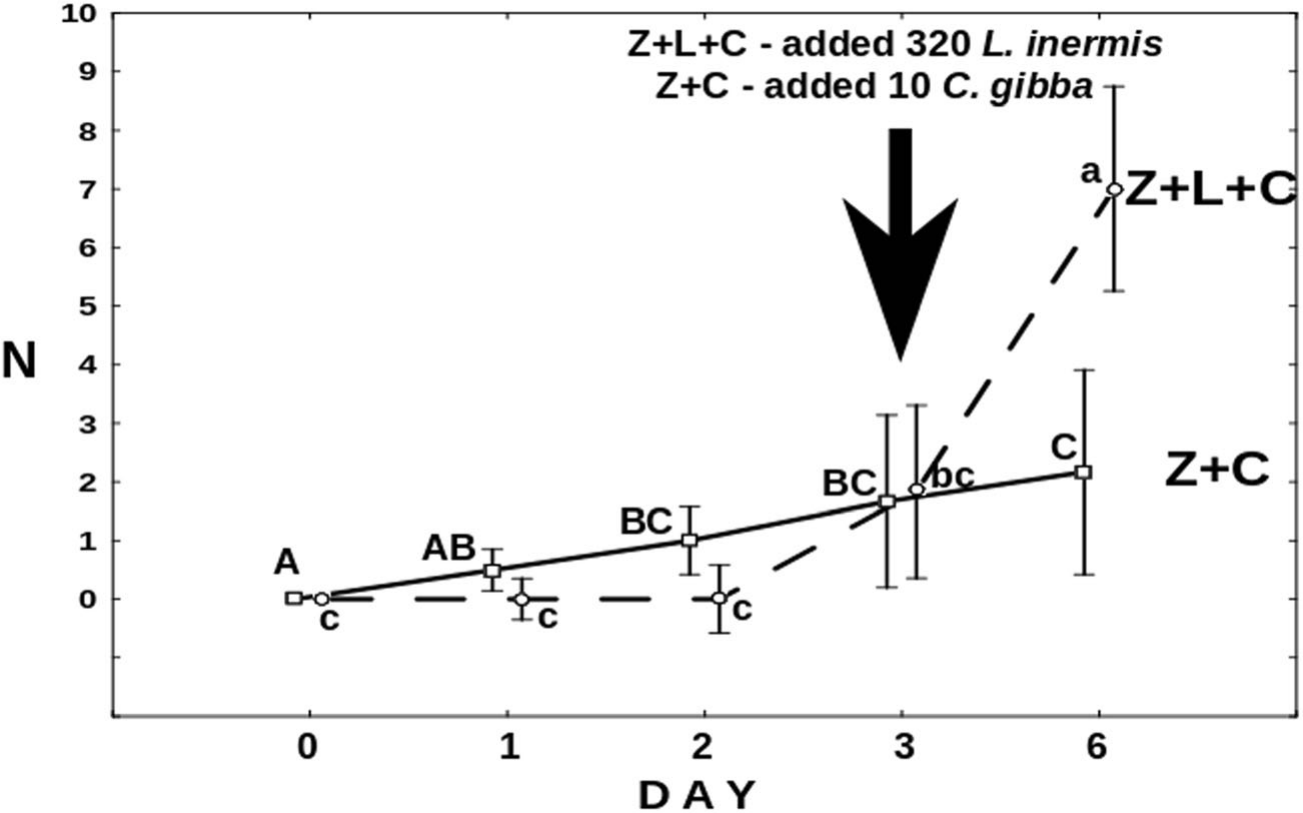
The mean number of *C*. *gibba* trapped by the fungus in the Z+L+C and Z+C treatments. Black arrow indicates the day of the inoculation of rotifers. Points sharing any common letter do not differ significantly (*p* > 0.05). Whiskers indicate confidence intervals

**Fig. 10:**
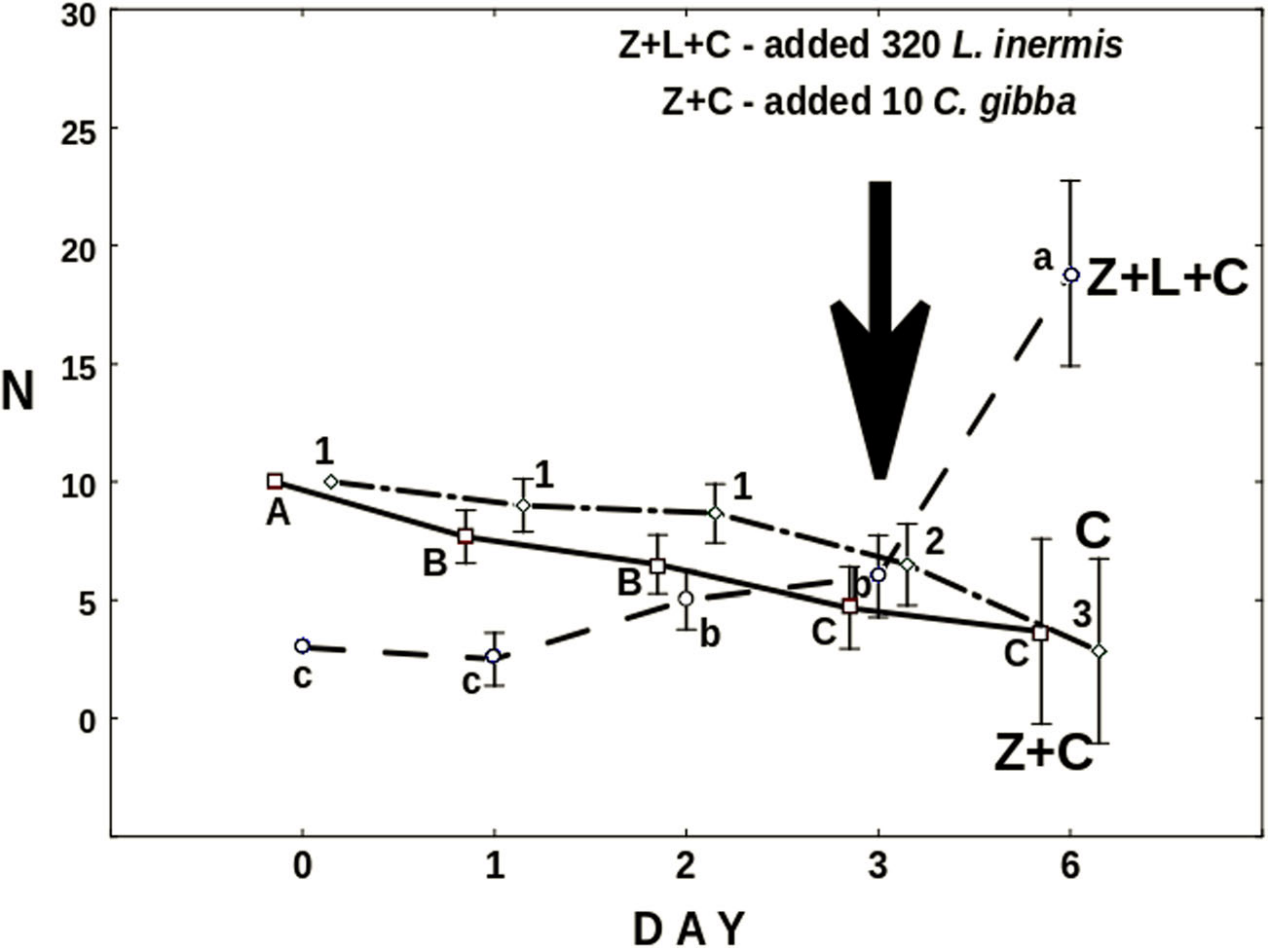
The mean number of active *C*. *gibba* in the Z+L+C and Z+C treatments and in the control (C) with only *C*. *gibba* present. Black arrow indicates the addition of 10 individuals of *C*. *gibba* into the Z+C treatment, and 230 of *L*. *inermis* to the Z+C+L treatment. Points sharing any common letter or number do not differ significantly (*p* > 0.05). Whiskers indicate confidence intervals

In the presence of *L*. *inermis*, approximately seven *Cephalodella* individuals per well were trapped by the fungus within 6 days (Fig. 9). The number of *Cephalodella* surviving in the presence of predatory fungus (Z+C) has been systematically decreasing although 10 fresh *Cephalodella* per well were supplied on the 3rd day of the experiment (Fig. 10). Interestingly, the changes in the number of active *Cephalodella* in the control treatment testing the effect of starvation on rotifer condition (C) were very similar to the ones just described. One has to bear in mind, however, that in the treatment with the both predators, additional number of *C*. *gibba* was added.

At the end of the experiment, the average number of active *Cephalodella* in the treatments Z+C and C approximated three, while the mean number of *Cephalodella* in the treatment Z+C+L reached 18 (Fig. 10).

**Fig. 11:**
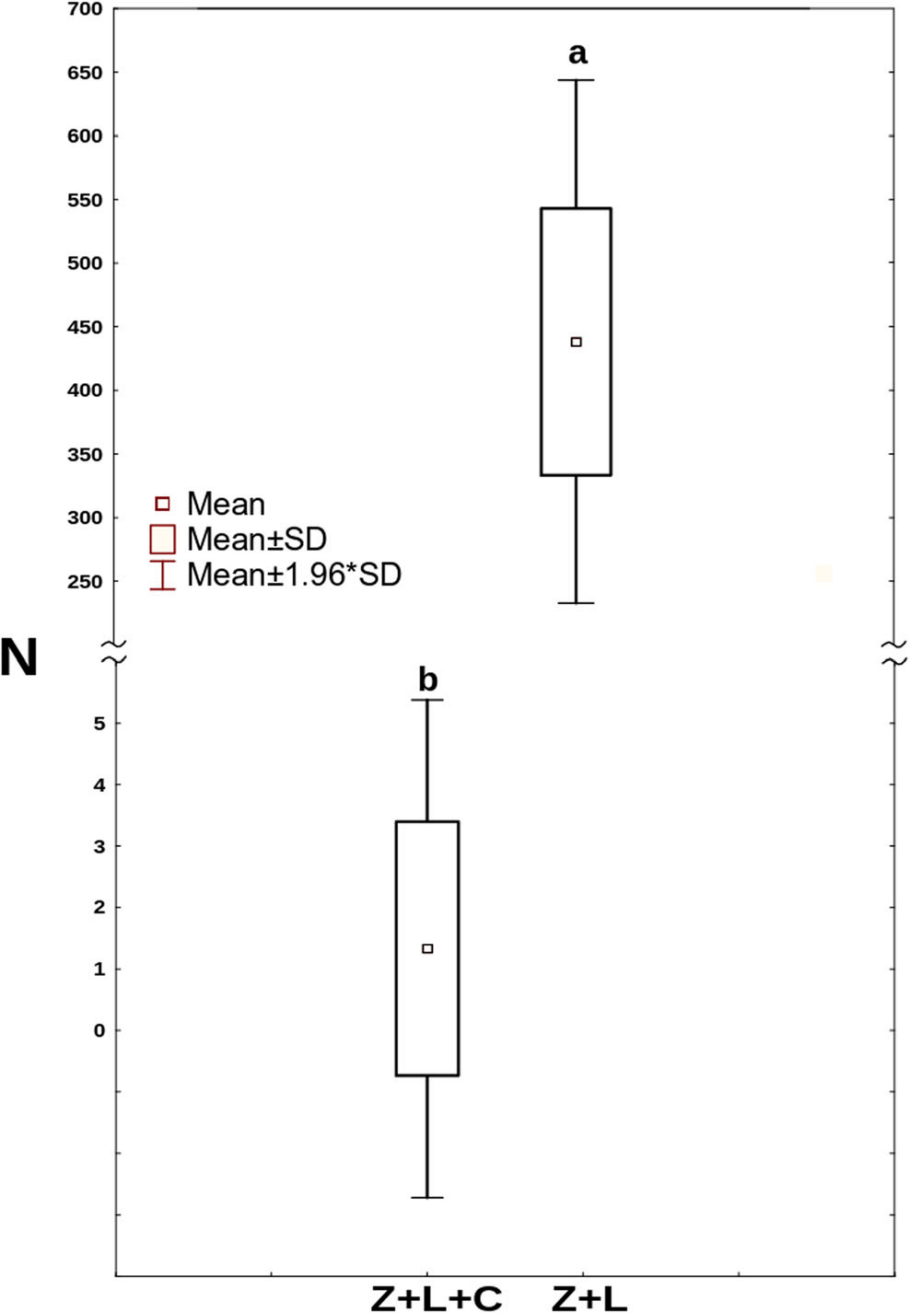
The mean number of *L*. *inermis* at the end of the experiment in treatment with both predators (Z+L+C) and with only the fungus (Z+L). Points sharing any common letter do not differ significantly (*p* > 0.05)

Although at the start of the experiment, the total number of *L*. *inermis* approximated 530, the joint presence of *Cephalodella* and conidia of the predatory fungus led to the almost total elimination of these rotifers. At the same time, the mean number of *L*. *inermis* in the treatment with conidia only exceeded 400 (Fig. 11).

## Discussion

Even though in researches of food webs competition and predation conducted in microcosm systems are often criticized as being oversimplification of much more complicated processes observed in nature, they provide an invaluable insight into ecological interactions which often are impossible to study in the field [35]. One of such complicated systems is activated sludge used in wastewater treatment. It is practically impossible to find two treatment plants with identical microbial communities. On the other hand, the same microorganisms such as rotifers, amoebae, or ciliates are found in most treatment plants. Even if not the same species, at least closely related organisms performing similar functions occur in activated sludge throughout the world. As our earlier research showed, also fungi preying on rotifers are quite common in treatment plants and all of them feed on *Lecane* rotifers [36].

Our first experiment showed strong impact of the top predator, the fungus, on the growth rate of an intermediate predator, *C*. *gibba*. When the latter had unlimited resources of *Lecane* rotifers, its population grew fast (Fig. 2). In the case when *Cephalodella* competed for *L*. *inermis* with the fungus, the number of its active individuals did not grow throughout the experiment. *C*. *gibba* quickly became extinct when it was the only food source for the fungus. Pimm and Lawton [37] suggested that when such an intermediate predator faces both predation and competition from the top predator, it has low chances of survival. However, as Polis and Holt [6] underline, there are many examples of such IGP interactions in natural habitats. In the case of our experiment, it also seems that even though competition for food apparently led to distinct limitation of *C*. *gibba* population, the rotifers persisted in the system. Schrag and Mittler [38] proved that, contrary to theoretical predictions, the coexistence of bacteria and bacteriophages was possible, thanks, among others, to the fact that the environment was less homogenous than previously thought. Taking into account how heterogonous the habitat of activated sludge is, it can be assumed that *C*. *gibba*, capable of exploiting resources other than *Lecane*, can still survive.

There is another factor which might contribute to rotifers’ survival. As was observed in the Z+L+C and Z+C treatments, at some point, the number of trapped rotifers stopped to rise, despite the fact that when *L*. *inermis* were available, the number of free-swimming *C*. *gibba* was rising (Fig. 3). The phenomenon might have resulted from the effect of fungus “saturation”—most of the fungus traps were blocked by trapped rotifers, both *C*. *gibba* and *L*. *inermis*.

Nevertheless, well-developed mycelium of *Zoophagus* can keep predatory rotifer population under control indirectly by limitation of its food source and directly by capturing it as a prey. As only few *Cephalodella* individuals were trapped by fungi in the Z+L+C treatment (Fig. 3), competition for food seems to have stronger effect on the rotifer population than predation. The impact of the presence of the top predator on intermediate consumer was also reflected in the number of eggs laid by *C*. *gibba* (Fig. 4) which was three times lower in the treatment where the fungus was present. Again, the fungus influenced the number of eggs in two ways: directly by killing *C*. *gibba* rotifers and indirectly by limiting their resources. The voracious fungus used in current experiments morphologically differs from earlier described species capable of catching rotifers, but its effectiveness in capturing *L*. *inermis* as a prey is as high as in the case of *Zoophagus* sp. [28] and *Lecophagus* sp. clones [39]. Rotifers are trapped by adhesive knobs of fungus usually by mouth region, then mycelium penetrates into the lorica, where it grows and digests rotifers’ tissues. As far as we know, there were no earlier observations of predatory fungus capturing *Cephalodella*.

Our experiments also showed the impact of two predatory organisms with completely different morphology and hunting strategy on the population of *L*. *inermis* rotifers. *C*. *gibba* actively searches for *Lecane*, rapidly captures them, and usually ingests the whole body (Supplementary Material - Mov 1). Taking into account that the initial number of *C*. *gibba* in the first experiment was only three individuals per well, it is astonishing that after 6 days only, solitary *L*. *inermis* remained, whereas the total number of prey provided during the experiment exceeded 950 individuals per well. When *L*. *inermis* was confronted with predatory fungus in a form of already developed mycelium, the result was similar to that when *Lecane* was under *C*. *gibba* pressure (Fig. 5). Only a few more rotifers survived in the presence of developed *Zoophagus* in comparison to the C+L treatment. Still, the number of remaining prey is extremely low in comparison with the total number of prey provided. The situation was similar when both predators competed with each other—*L*. *inermis* got almost extinct. It seems that in such an experimental IGP system, the prey does not have a chance to survive as both predatory organisms are really efficient in catching it. What is more, another interesting situation was observed in the course of experiments—*C*. *gibba* attacked and ingested *Lecane* already immobilized on a fungus trap and was able to move away unharmed by the fungus (Supplementary Material - Mov 2). As the first experiment was conducted on fungus already growing in form of long mycelium often protruding vertically, it was impossible to take reliable measurements. That is why the effect of intermediate consumer’s presence on fungus growth could not be quantitatively estimated.

The effect of the presence of the intermediate consumer on the top predator was investigated in the second experiment. During the first day, there were no differences between both treatments with *L*. *inermis* present (Fig. 7). As at that time there were no *C*. *gibba* trapped by the fungus (Fig. 9), the growth of the latter must have resulted from trapped *L*. *inermis*. Later on, the increase in mycelium length was higher in the absence of *C*. *gibba* (Fig. 7). Most probably, the intermediate consumer reduced the number of preys thus limiting the growth of the fungus. On the other hand, the number of trapped *C*. *gibba* was slowly growing throughout the experiment (Fig. 9) which probably stemmed from the fact that having surplus of prey *C*. *gibba* was proliferating (Fig. 10). In this context, it might seem strange that at the end of the experiment, the total length of mycelium in the treatment with both preys was almost three times lower than that in the treatment in which there were no *C*. *gibba* present; one may have assumed that if the fungus has an access to both preys, it should grow better. Most probably, however, *C*. *gibba*—a very effective competitor—rapidly eliminated vulnerable *L*. *inermis*, making it less accessible for young mycelium. Young predatory fungi grow faster without competitor, thanks to unlimited access to food.

Our experiment showed another interesting phenomenon. In the treatment where only 20 *Cephalodella* were provided as a sole food for conidia, the latter trapped solitary rotifers, but except for one conidium, they did not start to grow. Even the one growing eventually degenerated. The question why conidium captures *Cephalodella* if this does not result in the growth of mycelium remains unresolved. We could not exclude that, for some reason, *C*. *gibba* is not an adequate type of food for conidia. The fact that *C*. *gibba* was not a valuable food for the fungus may have strengthened the effect of slower grow of the fungus in the presence of both prey organisms; even if the mycelium was able to trap *C*. *gibba*, it still relied on *L*. *inermis* for growth. To make sure that after trapping a *C*. *gibba* the mycelium grows inside the rotifer, as it happens in the case of *L*. *inermis*, we used Calcofluor white staining, which clearly revealed mycelium growing inside *Cephalodella* body. As Polis and co-authors stated [7], intraguild predation involves not only killing competitors but also feeding on them. On the base of this definition, we can conclude that developed *Zoophagus* mycelium could be classified as intraguild predator. When conidia do not grow after trapping *Cephalodella*, such interaction should rather be defined as “interspecific killing” according to Fonseca and co-authors [40]. Even if a conidium of predatory fungus traps *C*. *gibba* but does not use it as a source of food, it eliminates a competitor.

The results of the second experiments confirmed that the chances of *L*. *inermis* rotifers’ survival under pressure of both predators are scarce. Almost all of 530 *Lecane* rotifers provided as a food in the Z+L+C treatment were eliminated, whereas in the treatment with sole fungi, over 400 *Lecane* individuals survived in the presence of a growing conidia. This strongly suggests that the preys were killed mostly by *Cephalodella*. Taking into account that at the beginning of the experiment in the Z+L treatment there were only 3 conidia per well, the effectiveness of fungus in catching rotifers is impressive. On average, over 100 individuals were eliminated during 6 days. This suggests that *Cephalodella* is a more effective competitor in comparison with the fungus. However, the advantage of *Cephalodella* is not surprising as *Zoophagus* needed time to develop mycelium which is more effective than conidia in catching rotifers, whereas *C*. *gibba* started to hunt immediately, quickly eliminating common prey. Although *Zoophagus* is able to catch *Cephalodella*, the result of competition between both predators depends mainly on quantitative relation between them. It is worth to underline that *Zoophagus* is an obligatory predator whereas *Cephalodella* can exploit other food sources. As it has been suggested, the presence of alternative food may promote coexistence of predators [10]. As shown by Finke and Denno [41], another factor strongly modifying IGP relations is the complexity of the habitat. Most probably, the habitat—in our case, activated sludge—which provides alternative food for intermediate consumer on the one hand, and numerous refuges for the prey on the other, is a perfect example of an environment in which the coexistence of organisms remaining in IGP relation can develop.

## Electronic Supplementary Material

Video 1 (M2TS 76848 kb)

Video 2 (M2TS 186996 kb)

## References

[1] Hairston NG, Smith FE, Slobodkin LB (1960). Community structure, population control and competition. Am Nat.

[2] Wardle DA, Yeates GW (1993). The dual importance of competition and predation as regulatory forces in terrestrial ecosystems: evidence from decomposer food-webs. Oecologia.

[3] Gurevitch J, Morrison JA, Hedges LV (2000). The interaction between competition and predation: a meta-analysis of field experiments. Am Nat.

[4] Chase JM, Abrams PA, Grover JP, Diehl S, Chesson P, Holt RD (2002). The interaction between predation and competition: a review and synthesis. Ecol Lett.

[5] Chesson P, Kuang JJ (2008). The interaction between predation and competition. Nature.

[6] Polis GA, Holt RD (1992). Intraguild predation: the dynamics of complex trophic interactions. Trends Ecol Evol.

[7] Polis GA, Myers CA, Holt RD (1989). The ecology and evolution of intraguild predation: potential competitors that eat each other. Annu Rev Ecol Syst.

[8] Root RB (1967). The niche exploitation pattern of the blue-gray gnatcatcher. Ecol Monogr.

[9] Arim M, Marquet PA (2004). Intraguild predation: a widespread interaction related to species biology. Ecol Lett.

[10] Marques RV, Sarmento RA, Oliveira AG, Rodrigues DDM, Venzon M, Pedro-Neto M (2018). Reciprocal intraguild predation and predator coexistence. Ecol Evol.

[11] Wissinger S, McGrady J (1993). Intraguild predation and competition between larval dragonflies: direct and indirect effects on shared prey. Ecology.

[12] Morin P (1999). Productivity, intraguild predation, and population dynamics in experimental food webs. Ecology.

[13] Löder MGJ, Boersma M, Kraberg AC, Aberle N, Wiltshire KH (2014). Microbial predators promote their competitors: commensalism within an intraguild predation system in microzooplankton. Ecosphere.

[14] Wilken S, Verspagen JM, Naus-Wiezer S, Van Donk E, Huisman J (2014). Biological control of toxic cyanobacteria by mixotrophic predators: an experimental test of intraguild predation theory. Ecol Appl.

[15] Miki T, Yamamura N (2005). Intraguild predation reduces bacterial species richness and loosens the viral loop in aquatic systems: ‘kill the killer of the winner’ hypothesis. Aquat Microb Ecol.

[16] Klimowicz H (1974). Biological studies of the sewage-treatment processes in the city of Toruń. Pol Arch Hydrobiol.

[17] Saratovskikh EA, Kozlova NB, Papin VG, Shtamm EV (2006). Biochemical and photochemical degradation of the herbicide Lontrel. Appl Biochem Microbiol.

[18] Fiałkowska E, Pajdak-Stós A (2008). The role of Lecane rotifers in activated sludge bulking control. Water Res.

[19] Fiałkowska E, Kocerba W, Pajdak-Stós A, Klimek B, Fyda J (2011). Clonal variation in reproductive response to temperature by a potential bulking control agent, *Lecane inermis* (Rotifera). Water Sci Technol.

[20] Pajdak-Stós A, Kocerba W, Fiałkowska E, Klimek B, Fyda J (2011). The effect of medium on selected life-history traits in three clones of *Lecane inermis* (Rotifera) from activated sludge. Water Sci Technol.

[21] Kocerba-Soroka W, Fiałkowska E, Pajdak-Stós A, Klimek B, Kowalska E (2013). The use of rotifers for limiting filamentous bacteria Type 021N, a bacteria causing activated sludge bulking. Water Sci Technol.

[22] Sobczyk M, Fiałkowska E, Pajdak-Stós A, Fyda J (2013) Effect of *Lecane inermis* rotifers grazing on wastewater bacteria biofilms. 2nd Young Scientists Conference, World Water Day, Poznań 10.13140/RG.2.1.4080.5361

[23] Drzewicki A, Kowalska E, Pajdak-Stós A, Fiałkowska E, Kocerba-Soroka W, Sobczyk Ł (2015). Experimental attempt at using *Lecane inermis* rotifers to control filamentous bacteria Eikelboom type 0092 in activated sludge. Water Environ Res.

[24] Wacker A, Weithoff G (2009). Carbon assimilation mode in mixotrophs and the fatty acid composition of their rotifer consumers. Freshw Biol.

[25] Schmid-Araya JM, Schmid PE (1995). Preliminary results on diet of stream invertebrate species: the meiofaunal assemblages. Jahresbericht Biologische Station Lunz.

[26] Whisler HC, Travland LB (1974). The rotifer trap of Zoophagus. Arch Microbiol.

[27] Glockling SL (1997). *Zoophagus cornus*: a new species from Japan. MycolRes.

[28] Pajdak-Stós A, Ważny R, Fiałkowska E (2016). Can a predatory fungus (Zoophagus sp.) endanger the rotifer populations in activated sludge?. Fungal Ecol.

[29] Pajdak-Stós A, Fiałkowska E, Fyda J, Kocerba-Soroka W, Sobczyk M (2017) A method of mass culture of Lecane rotifers European Patent EP 14731401.7

[30] Nogrady T, Pourriot R, Nogrady T, Pourriot R, Segers H, Rotifera (1995). The Notommatidae. The Notommatidae and the Scaridiidae. Guides to the identification of the microinvertebrates of the continental waters of the world.

[31] Segers H, Dumont HJF (1995). The Lecanidae (Monogononta), Rotifera. Guides to the identification of the microinvertebrates of the continental waters of the world.

[32] Dick MW (1990). The systematic position of *Zoophagus insidians*. Mycol Res.

[33] Crawley MJ (2002). Statistical computing. An introduction to data analysis using S-Plus.

[34] StatSoft, Inc. (2014) STATISTICA (data analysis software system), version 12. www.statsoft.com

[35] Jessup CM, Kassen R, Forde SE, Kerr B, Buckling A, Rainey PB, Bohannan BJ (2004). Big questions, small worlds: microbial model systems in ecology. Trends Ecol Evol.

[36] Fiałkowska E, Pajdak-Stós A, Starzycka J (2016). The diversity of fungi preying onrotifers in wastewater treatment plants.

[37] Pimm SL, Lawton JH (1978). On feeding on more than one trophic level. Nature.

[38] Schrag SJ, Mittler JE (1996). Host-parasite coexistence: the role of spatial refuges in stabilizing bacteria-phage interactions. Am Nat.

[39] Fiałkowska E, Pajdak-Stós A (2018). Temperature-dependence of predator-prey dynamics in interactions between the predatory fungus *Lecophagus* sp. and its prey *L. inermis* rotifers. Microb Ecol.

[40] Fonseca MM, Montserrat M, Guzmán C, Torres-Campos I, Pallini A, Janssen A (2017). How to evaluate the potential occurrence of intraguild predation. Exp Appl Acarol.

[41] Finke DL, Denno RF (2006). Spatial refuge from intraguild predation: implications for prey suppression and trophic cascades. Oecologia.

